# SiC/MoSi_2_-SiC-Si Oxidation Protective Coatings for HTR Graphite Spheres with Residual Si Optimized

**DOI:** 10.3390/ma15093203

**Published:** 2022-04-28

**Authors:** Xiaoyu Wei, Hui Yang, Hongsheng Zhao, Xiaoxue Liu, Kaihong Zhang, Ziqiang Li, Yuan Gao, Bing Liu

**Affiliations:** Institute of Nuclear and New Energy Technology, Tsinghua University, Beijing 100084, China; wei-xy@outlook.com (X.W.); yang-hui@mail.tsinghua.edu.cn (H.Y.); liuxx1@mail.tsinghua.edu.cn (X.L.); zkh@tsinghua.edu.cn (K.Z.); liziqiang@tsinghua.edu.cn (Z.L.); gy1998@mail.tsinghua.edu.cn (Y.G.); bingliu@mail.tsinghua.edu.cn (B.L.)

**Keywords:** anti-oxidation, C/SiC, MoSi_2_, coating, nuclear graphite

## Abstract

SiC/MoSi_2_-SiC-Si coatings for nuclear graphite spheres with different Si-Mo ratios were prepared through two-step pack cementation. XRD, SEM and EDS techniques were used to analyze the composition and microstructure of the coatings. The oxidation resistance performance of the composites at 1773 K, in static air, was investigated. The results showed that the SiC-MoSi_2_-Si coating could be divided into a denser inner layer and a loose outer layer, as free Si would infiltrate into the inner micropores of the coating under capillary force. When the Si/Mo ratio of the second pack cementation was 7:1, the thickness of the denser inner layer basically reached the maximum and exhibited excellent oxidation resistance ability, with a weight gain of 0.19% after 200 h oxidation. The performance improvement was analyzed as a result of the addition of SiC and C powder in the pack cementation process, effectively increasing the phase interfaces to relax the thermal stress in the coating. With different Si-Mo ratios, the content of residual Si and the formation rate of SiO_2_ glass layer on the coating surface were also different, thus affecting the anti-oxidation performance. The main reactions occurring at different stages of the oxidation curve were also discussed.

## 1. Introduction

Graphite material has excellent properties, such as low expansion coefficient, high thermal conductivity, good thermal stability and high specific strength, etc., ensuring its application in the manufacture of important devices in high and ultra-high temperature environments [1,2,3,4,5]. Besides, in the field of nuclear energy, due to the advantages of a low neutron absorption cross-section and suitable isotropy, graphite is an important part of the spherical fuel element in high temperature gas-cooled reactors (HTGR) [6,7,8,9,10,11]. However, under the oxidative atmospheres with temperatures above 773 K, graphite is easily oxidized, losing its excellent mechanical properties, which greatly limits its practical application effect [12,13,14].

Covering the surface of graphite material with a sealing and insulating coating, especially a multi-layer coating, can effectively slow down the oxidizing substances’ penetration to the surface of the graphite substrate [14]. Therefore, various coating materials have been developed for oxidation protection up to now. Among them, MoSi_2_ has a high melting point (2303 K), a suitable density (6.24 g/cm^3^), and excellent oxidation resistance at high temperatures, demonstrating great potential in high temperature structural materials [15,16]. However, the thermal expansion coefficient of MoSi_2_ (7.8 × 10^−6^ K^−1^) does not match the graphite substrate (4.0 × 10^−6^ K^−1^); therefore, it is not suitable to be directly coated on the graphite surface. SiC can be used to bond graphite substrates and outer coatings due to its superior physical and chemical adaptability of its coating-to-matrix and bonding layer-to-outer layer [14,17]. In 2006, Liu et al. [16] used SiC/Si-MoSi_2_ coating to improve the oxidation resistance of carbon materials, and the weight loss was 0.36% in 100 h at 1673 K, which preliminarily verified the feasibility of SiC/MoSi_2_ for anti-oxidation coating.

On this basis, researchers further enhanced the anti-oxidation properties of the coating by introducing other elements or optimizing the Si-Mo ratio [14,18,19,20,21,22,23,24]. The SiC/SiC-Mo-W coating prepared by Fu et al. by introducing W metal could only lose 0.003% in weight when exposed to air at 1673 K for 220 h and gained 1.56% in 252 h at 1773 K [23]. Zhang et al. [20] obtained a multi-layer coating of SiC/Si-Mo-B by introducing a B element. The weight loss of 300 h oxidation at 1873 K was 0.65%, while the weight loss without the B element was 3.25%. Based on SiC/Si-Mo coating, Zhang et al. [21] studied the effect of the Mo/Si ratio on the oxidation resistance of the coating. At the optimal ratio (0.2 Mo/Si ratio), 1773 K, 103 h, the weight loss was 2.47%.

However, there are still some problems in these current studies. Firstly, most of them only changed the selection of materials, and seldom explored the anti-oxidation mechanism in-depth, which has little inspiration for follow-up research [20,22,23]. Besides, although current research has made a certain improvement in the oxidation resistance performance, potential still exists for simple SiC/MoSi_2_ multilayer coatings. According to the research of Zhou et al. [7], by the two-step pack cementation method, a spherical graphite matrix was coated with SiC/Si coating. Then after oxidation in high temperature static air of 1773 K for 200 h, the weight gain of the sample was only 0.52%, which was superior to the performance of the majority of current SiC/Si-Mo based coatings. Therefore, in theory, better oxidation resistance could be achieved by changing the experimental conditions and optimizing the free Si content in the preparation of SiC/Si-Mo coating.

In this paper, on the basis of the research of Zhou et al. [7], Mo powder was added in the second pack cementation process, and the weight gain of the obtained samples at 1773 K for 200 h ranged from 0.19% to 0.56%, which was an obvious improvement compared with the previous Si-Mo based anti-oxidation coatings [13,20,21,22,23]. Combining SEM images and EDS analysis, the microscopic morphology of SiC-MoSi_2_-Si coating appeared layered. As molten free Si infiltrated inward under capillary force at high temperature, the inner layer was denser, while the outer layer was slightly looser, with the content of MoSi_2_ relatively higher. The thickness of the inner layer could be increased by increasing the Si/Mo ratio. When the Si/Mo ratio was 7:1, the thickness of the inner layer basically reached the maximum, and the oxidation resistance was the best at this time, with only 0.19% weight gain at 200 h. By analyzing the oxidation curves, the main reactions occurring at different oxidation stages were studied, and combined with the SEM results to analyze the effect of residual Si on the antioxidant properties. In conclusion, better oxidation resistance performance without introducing new elements was achieved, which simplified the experimental operation and had more enlightening significance for the SiC/Si-MoSi_2_-based system.

## 2. Experiment

The matrix graphite spheres used in the experiment were taken from the HTR fuel element produced by the Institute of Nuclear and New Energy, Tsinghua University, with a diameter of 15 mm. The density of graphite was 1.7–1.73 g/cm^3^, the thermal conductivity was higher than 30 W/(m·K) (1273 K), and the anisotropy of thermal expansion α_┴_</α_//_ ≤ 1.3. The powders used are purchased from Beijing Xingrongyuan Technology Co., Ltd. (Beijing, China) The mass fraction of the powders for pack cementation in the first step is 60% Si, 20% SiC, 10% natural graphite powder and 10% Al_2_O_3_. Graphite sintering furnace ZT-40-20 is produced by Shanghai Chenhua Science Technology Co., Ltd. (Shanghai, China) Using a graphite sintering furnace, the samples were subjected to high temperature treatment at around 1973 K under the protection of Ar gas, thereby obtaining the inner SiC coating. Subsequently, the samples were polished on a grinding and polishing machine at 300 rpm/min for 15 min with 300-grit sandpaper, and after ultrasonic cleaning, vacuum-dried at 373 K for 12 h. The powder for pack cementation in the second step was composed of Si, Mo, Al_2_O_3_ and natural nuclear graphite powder. The mass ratios of Si and Mo were changed to 1:1, 3:1, 5:1 and 7:1 respectively, as shown in Table 1. The samples were subjected to high temperature treatment in a vacuum environment around 2173 K to obtain the outer layer SiC/MoSi_2_/Si coating. After ultrasonic cleaning, the samples were vacuum dried at 373 K for 12 h.

Surfaces and cross-sections of the samples were observed by scanning electron microscopy (SEM, Quanta 200 FEG, FEI, Eindhoven, The Netherlands). Molecular structures were analyzed by Raman spectroscopy (LabRAM HR Evolution, Horiba Jobin Yvon, Paris, France). The phases of the coatings were analyzed by X-ray diffractometer (XRD, model D/Max IIIA; Rigaku, Tokyo, Japan). The sample preparation method for XRD detection was as follows: the coated graphite pebbles were first broken, and then oxidized in air at 1073 K for 10 h in a muffle furnace to completely consume the graphite. The oxidation weight loss of the coating at 1773 K was tested by a muffle furnace to characterize its oxidation resistance ability.

## 3. Results and Discussion

### 3.1. Microstructure

**Figure 1:**
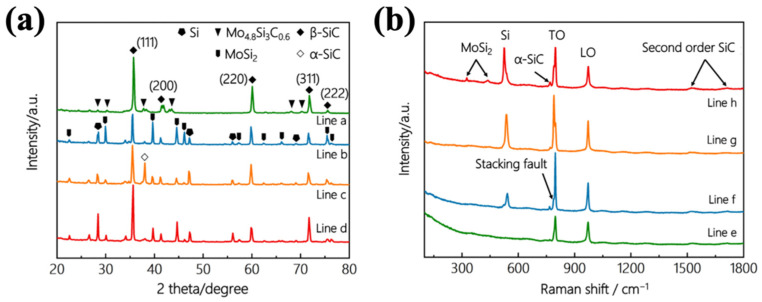
(**a**) XRD patterns of the SiC/MoSi_2_-SiC-Si coatings with different Si/Mo ratio: a, 1:1; b, 3:1; c, 5:1; d, 7:1. (**b**) Raman patterns of the coatings with different Si/Mo ratio: e, 1:1; f, 3:1; g, 5:1; h, 7:1.

Figure 1a was the XRD pattern of the as-prepared coating. It could be seen from the figure that the coating was mainly composed of SiC (especially β-SiC), MoSi_2_, free Si, and a small amount of Mo_4.8_Si_3_C_0.6_ (when Si-Mo ratio was 1:1, excess Mo reacted with Si and C). SiC was generated from the reaction of Si and C, in addition to the SiC powder directly added during the first pack cementation. MoSi_2_ was obtained by the reaction of Si and Mo in the second pack cementation, and excess Si existed in free form. The Raman spectrum results were shown in Figure 1b. The main phase of the SiC crystal was β-SiC with a cubic structure. It had a zone-center transversal optical phonon (TO) mode at 796 cm^−1^ and a zone-center longitudinal phonon (LO) mode at 972 cm^−1^. The low-intensity peaks in the range of 1450~1700 cm^−1^ were ascribed to the second-order scattering of the acoustic signal of SiC. The Si crystal signal was detected at 524 cm^−1^, and the MoSi_2_ crystal signal was detected at 430 cm^−1^ and 325 cm^−1^. These results were basically consistent with the XRD results.

**Figure 2:**
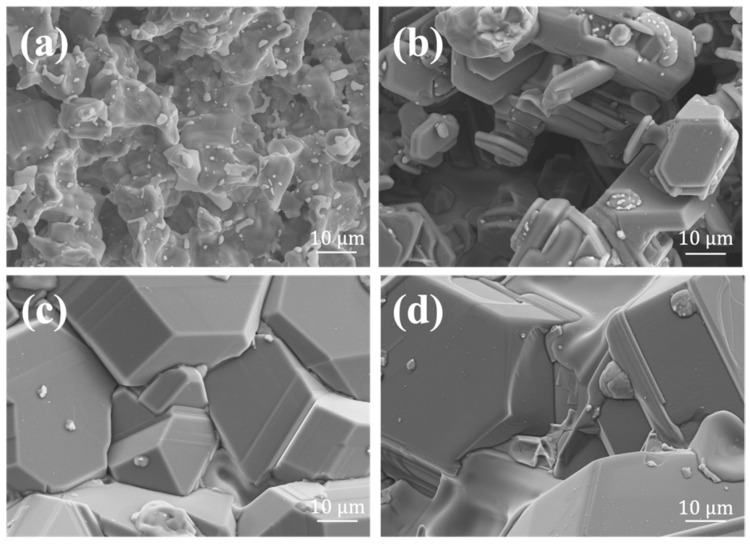
SEM images of the surface of SiC/MoSi_2_-SiC-Si coatings with different Si/Mo ratio: (**a**) 1:1, (**b**) 3:1, (**c**) 5:1, (**d**) 7:1.

Figure 2 was the SEM images of the surfaces of the Si-Mo coatings. It could be seen that with the increase in the Si-Mo ratio, the coating surface became denser and denser. When the Si-Mo ratio was 1:1, the surface was very loose, and no large grains were formed. When the Si-Mo ratio was 3:1, although grains appeared on the surface, the size was small, and there were still obvious defects. When the Si-Mo ratio was 5:1 or 7:1, the cracks on the surface basically disappeared, showing a denser morphology.

**Figure 3:**
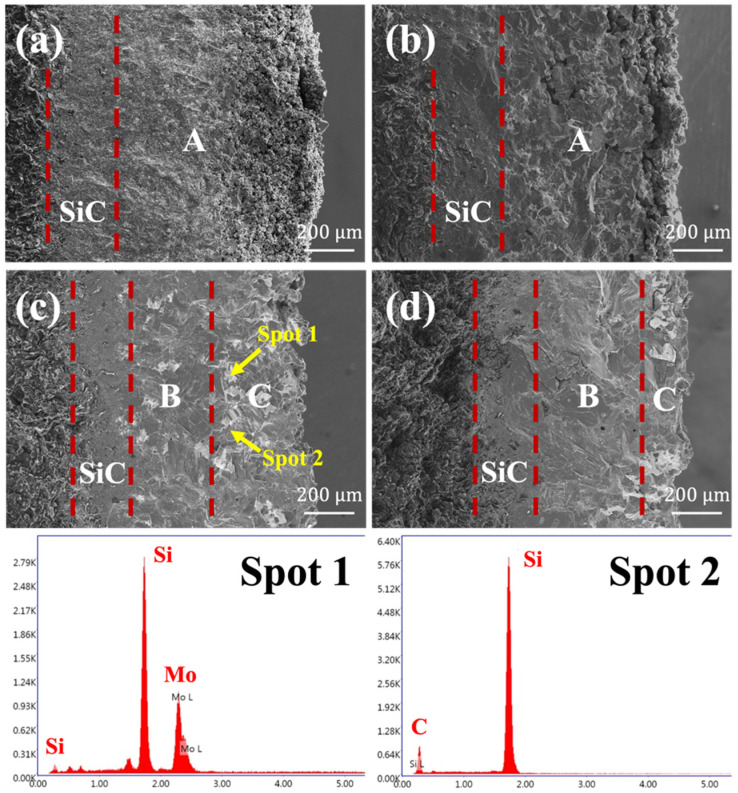
SEM images of the cross-section of SiC/MoSi_2_-SiC-Si coatings with different Si/Mo ratio: (**a**) 1:1, (**b**) 3:1, (**c**) 5:1, (**d**) 7:1.

The microstructure of the coatings’ cross-section was shown in Figure 3. The thickness of the coatings with different compositions was similar, and the overall thickness was about 1 mm. There was good adhesion between the inner coating and the graphite substrate, and also between the outer coating and the inner coating, with no obvious boundary. According to the results of EDS, the white part in the image was dominated by MoSi_2_ and free Si phase, while the dark part was SiC and free Si phase. The four coatings all had a relatively uniform dark layer on the inner side closest to the graphite substrate, representing the inner SiC coating produced by the first pack cementation. However, as the Si-Mo ratio increased, the thickness of the loose part of the outermost layer gradually decreased, and the structure of the external coating introduced by the second pack cementation was quite different. During the second embedding, Si, Mo, and C powder reacted under vacuum at 2173 K to generate MoSi_2_ and SiC. When the Si-Mo ratio was 1:1, the molar ratio of Si-C-Mo was 3.4:2.4:1. At this time, the amount of Si was not enough to fully react with C and Mo, thus the external coating had only a small amount of unreacted free silicon, leaving many voids as a whole, as shown in Figure 3a. When the Si-Mo ratio was increased to 3:1, the molar ratio of Si-C-Mo was 10.3:4.9:1. The overall content of unreacted Si in the outer coating was still relatively small, so although it was much denser than Si-Mo 1:1, still distributed with many tiny holes. When the Si-Mo ratio was further increased to 5:1, the content of residual Si in the outer coating was further increased. At this time, since the embedding and reacting process was carried out under a vacuum, the gas pressure in the tiny pores of the coating was as low as 100 Pa [25]. With liquid free Si infiltrating into the pores under capillary force, obvious delamination appeared in the external coating, as shown in Figure 3c. The inner B region was much denser and the content of the white MoSi_2_ phase was relatively lower, while the outer C region was slightly loose, with more MoSi_2_ phase. The thickness of the two regions was basically the same. When the Si content was further increased, as shown in Figure 3d (Si-Mo 7:1), the thickness of the B region further increased, that is, free Si filled a thicker range, and the coating overall became much denser.

### 3.2. Oxidation Resistance

**Figure 4:**
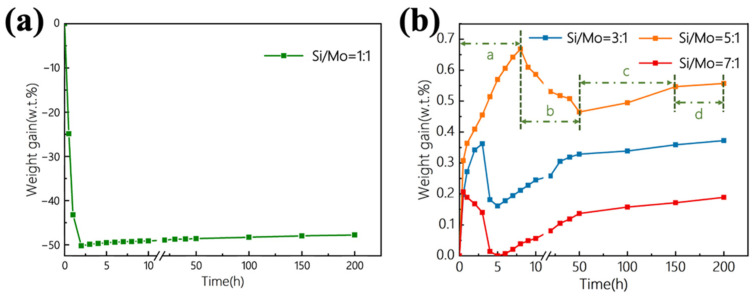
Isothermal oxidation curves of the coated samples with different Si/Mo ratio: (**a**) 1:1; (**b**) 3:1, 5:1, 7:1.

The oxidation weight loss curves of the graphite materials protected by coatings with different Si-Mo ratios in static air at 1773 K were shown in Figure 4. When the Si-Mo ratio was 1:1, the graphite material lost 50% of the weight in about 2 h, which was consistent with the loose and porous coating structure observed earlier. When the Si/Mo ratios were 3:1, 5:1 and 7:1, the weight gain after 200 h oxidation was 0.37%, 0.56% and 0.19%, respectively, that is, the oxidation resistance performance of 7:1 was the best. The weight gain curve could be roughly divided into 4 stages, as shown in Figure 4b. There was a rapid weight gain process (a) in the early stage of oxidation, followed by a weight loss process (b). The duration of these two processes was quite short, ranging from several to dozens of hours. After that, the sample would gain weight a bit (c) and then more slowly (d).

A series of reactions that could occur in the coating in static air at 1773 K were shown in Table 2. There was inevitably a small amount of oxygen in the graphite ball, which would oxidize graphite to generate CO gas at high temperatures. CO reacted with Si, that was, reaction (1) [26]. However, due to the low CO content, this reaction had little effect on the mass change. In the early stage of oxidation, oxygen diffused into the coating through surface defects and contacted and reacted with free Si, SiC, and MoSi_2_. The oxidation rate was controlled by the reaction rate. As the activation energy of Si was lower, the initial oxidation process was dominated by reaction (2). Therefore, the oxidation curve exhibited an obvious weight gain in segment (a). When the Si near surface was oxidized, the generated SiO_2_ would fill part of the micro-cracks, making the coating denser and reducing the penetration of oxygen. However, the oxidation of SiC and MoSi_2_ near the surface would continue, mainly (4)–(6), and the oxidation curve showed a slight weight loss in the (b) segment. When a complete SiO_2_ glass layer was formed inside and on the surface of the coating, oxygen needed to pass through the glass layer to react with the internal substances. The oxidation rate was determined by the oxygen diffusion rate. Reactions (1)–(6) all occurred, and the oxidation curve showed a gradually slower weight gain in parts (c) and (d).

Among the samples, when the Si-Mo ratio was 7:1, the oxidation resistance performance was the best, with weight gain of only 0.19% at 1773 K for 200 h. Besides, the weight gain was stable and slow in the second half of the oxidation curve, indicating that this kind of coating could protect the graphite substrate from being oxidized for more than 200 h. Even the worst-performing sample with a Si-Mo ratio of 5:1 among the three ratios, the weight gain at 200 h was only 0.56%, which also reflected excellent oxidation resistance ability. Comparing the results in the previous works, whether it was a two-step pack cementation of pure SiC or adding new elements to the Si-Mo coating [12,13,14,15,16,18,19,20], the SiC/MoSi_2_-SiC-Si coating in this work performed better on oxidation resistance. The difference was mainly due to the different compositions of the powder during embedding. In the first pack cementation, the pack cementation powders were composed of 60% Si, 20% SiC, and 10% Si, while SiC powder was not usually used in other Si-Mo coating studies [20,21,22,23]. In this work, the added SiC powder and the reacted SiC could form a multi-phase microstructure, increasing the phase interface to relax the thermal stress in the coating [14]. In addition, even considering that Si would infiltrate into the graphite substrate for reaction, the amount of Si used was obviously to excess, and the residual Si could ensure the relative dense structure in the coating of the first pack cementation. During the second pack cementation, Si, Mo, and C were added, while few other studies had used C powder. C powder could effectively reduce the mismatch of thermal expansion coefficient between the inner coating and the outer coating [13], which would also reduce the cracks generated during the heating and cooling process of the coating, thereby improving the oxidation resistance of the coating. The reason for the better performance of the SiC/MoSi_2_-SiC-Si coating than the pure SiC coating prepared by the same method was the existence of MoSi_2_. The presence of the MoSi_2_ phase could increase the phase interface in the coating, which was beneficial to prevent the propagation of cracks. Moreover, MoSi_2_ had the properties of high strength and high melting point, which means it had a pinning effect during the oxidation process, protecting the structural integrity and stability of the coating. In addition, in the process of repeated heating and cooling, when the temperature was around 1273 K, MoSi_2_ would undergo a brittle-ductile transition. This process could absorb microcracks and residual stress in the coating, thereby improving the thermal shock resistance and fracture toughness of the coating.

As for the differences in coatings with different Si-Mo ratios, the main cause was the difference in the content of free Si. The three curves in Figure 4b were quite different from each other in segment (a), while the difference between the subsequent weight loss and weight gain stages was little. According to the previous discussion, the main reaction in segment (a) was the oxidation of free Si. When the Si-Mo ratio was 3:1, the free Si content was relatively low, so the oxidized amount was small, that is, the weight gain was small in the initial stage of oxidation. When the Si-Mo ratio was 5:1, the free Si content was slightly higher, as shown in Figure 3c, filling the inner side of the SiC-MoSi_2_-Si coating. However, the content of free Si at the outer surface of the coating was still relatively low, making it slower to form a complete and continuous SiO_2_ glass layer on the surface. Oxygen continued to infiltrate into the coating and reacted with free Si inside. Therefore, as reflected in Figure 4b, the segment (a) was nearly 10 h. When the Si-Mo ratio was 7:1, the free Si content was more, basically enough to fill the SiC-MoSi_2_-Si coating, which means a complete SiO_2_ glass layer could quickly appear when it was oxidized. By greatly slowing down the rate of oxygen penetration into the coating, the oxidation rate of the entire sample was slowed down either, corresponding to the excellent anti-oxidation property of this coating. If the content of Si continued to increase, it was easy to generate holes due to the volatilization of Si at high temperatures (at 1773 K, the vapor pressure of Si is 1300 Pa) [27], and the excess Si would also melt and flow out before being oxidized, making more coating defects exposed in the air and oxidizing the internal graphite substrate quickly. Therefore, when the Si-Mo ratio was 7:1, the free Si content was believed to be the most reasonable, which could not only fill the pores inside the coating but also would not accelerate the aging and damage of the coating due to excess.

**Figure 5:**
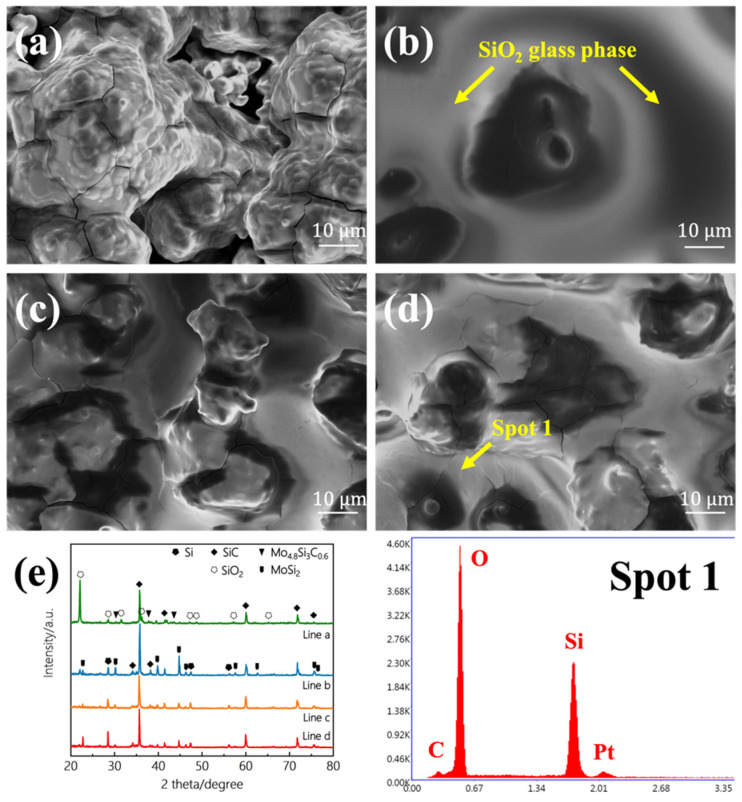
SEM images of the surface of SiC/MoSi_2_-SiC-Si coatings after oxidation with different Si/Mo ratio: (**a**) 1:1, (**b**) 3:1, (**c**) 5:1, (**d**) 7:1. (**e**) XRD patterns of the SiC/MoSi_2_-SiC-Si coatings after oxidation with different Si/Mo ratio: a, 1:1; b, 3:1; c, 5:1; d, 7:1.

The surface of the oxidized coating was shown in Figure 5. According to the results of XRD and EDS, the SiO_2_ glass phase was formed on the surfaces of all four different coated samples. When the Si-Mo ratio was 5:1 and 7:1, a complete SiO_2_ glass layer was formed, and the number of cracks of 5:1 was slightly larger. When Si-Mo was 3:1, the SiO_2_ glass layer on the surface was not complete and had penetrating cracks, which was consistent with the insufficient free silicon content analyzed above.

**Figure 6:**
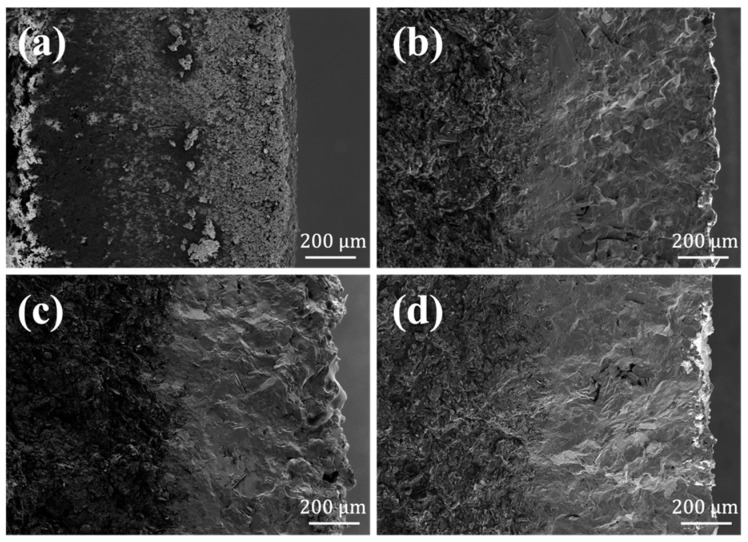
SEM images of the cross-section of SiC/MoSi_2_-SiC-Si coatings after oxidation with different Si/Mo ratio: (**a**) 1:1, (**b**) 3:1, (**c**) 5:1, (**d**) 7:1.

Figure 6 was the SEM images of the cross-section of the oxidized coating. Apart from the Si-Mo ratio of 1:1, which has a loose microstructure, penetrating cracks did not appear in the rest of the coatings. Only several defects appeared in the cross-section, mainly due to the thermal expansion coefficient difference in the heating and cooling process. If the coating was heated up again, the molten Si was able to fill these cracks, and the oxidation resistance ability would not be affected. These images verified once again that the coatings in this research had played a good protective role and did not fail in high temperature air.

## 4. Conclusions

By changing the composition of the embedded powder, a SiC/MoSi_2_-SiC-Si coating with better oxidation resistance ability was prepared, which had a weight gain of only 0.19% in static air of 1773 K for 200 h, demonstrating the application potential in oxidative protection for HTR Graphite spheres. The improvement in anti-oxidation performance was mainly due to the use of SiC and C powders. By adjusting the Si-Mo ratio and analyzing it with SEM and EDS methods, the SiC-MoSi_2_-Si coating had obvious delamination with the change of free Si content. The higher the free Si content, the thicker the dense inner layer. When the Si-Mo ratio was 7:1, the inner layer was basically the thickest and the oxidation resistance performance was also the best. The oxidation process could be divided into four stages: rapid mass increase, mass decrease, slow mass increase, and stable mass.

## Figures and Tables

**Table 1:** Detailed composition of the powder used in the second pack cementation process.

Si/wt%	Mo/wt%	C/wt%	Al_2_O_3_/wt%	Si/Mo Mass Ratio	Si/C/Mo Molar Ratio
42.5	42.5	13	2	1:1	3.4:2.4:1
64	21			3:1	10.3:4.9:1
71	14			5:1	17.1:7.4:1
74.4	10.6			7:1	23.9:9.8:1

**Table 2:** Reactions and their Gibbs free energy during the oxidation process in SiC-MoSi_2_-Si coatings in 1773 K air.

Reaction No.	Reaction Equation	∆G^θ^/(kJ·mol^−1^)
(1)	2Si + CO (g) = SiC + SiO (g)	−32.488
(2)	Si + O_2_ (g) = SiO_2_	−595.800
(3)	2SiC + 3O_2_ (g) = 2SiO_2_ + 2CO (g)	−807.984
(4)	SiC + O_2_ (g) = SiO (g) + CO (g)	−456.856
(5)	2SiC + 3O_2_ (g) = SiO (g) + 2CO_2_ (g)	−1172.173
(6)	2MoSi_2_ + 7O_2_ (g) = 2MoO_3_ (g) + 4SiO_2_	−1283.830

## Data Availability

The data presented in this study are available in the article.
